# 476. Epidemiology and Outcomes of Candidemia in a Large Academic Medical Center

**DOI:** 10.1093/ofid/ofac492.534

**Published:** 2022-12-15

**Authors:** Zachary Pek, Jacqueline T Bork, John W Baddley

**Affiliations:** University of Maryland Medical Center, Baltimore, Maryland; University of Maryland School of Medicine, Severna Park, Maryland; University of Maryland Medical Center, Baltimore, Maryland

## Abstract

**Background:**

Candidemia is an important cause of morbidity and mortality. Fluconazole resistance is associated with increased mortality in candidemia and thus echinocandins are recommended as first line empiric therapy. To understand the current landscape of candidemia treatment, we evaluated the epidemiology and outcomes of candidemia at our medical center, with a focus on differences in traditional azole-susceptible and non-susceptible candida species.

**Methods:**

This is a retrospective cohort study including unique episodes of candidemia from 7/2017 – 12/2021 in hospitalized adult patients. We characterized candidemia episodes by species, age, hospital-onset, hospital location and critical illness. We compared traditionally azole susceptible organisms (e.g. C.albicans, C. parapsilosis, C.tropicalis) to non-susceptible (e.g. C.krusei, C.glabrata) based on antibiogram (cut off >94% susceptible) with the primary outcome of 30-day mortality. Secondary outcomes were recurrent/persistent candidemia, 90-day mortality and length of stay. Comparisons were done with χ2 or Wilcoxon rank sum tests. Logistic regression was performed to determine if azole-nonsusceptible candidemia was associated with 30-day mortality.

**Results:**

404 candidemic episodes for 389 patients were identified, of which 72% were due to azole-susceptible Candida spp. and C.glabrata and C.krusei were 23% and 5%, respectively. Critically ill patients were more likely to have azole-non-susceptible Candida spp (59 vs. 48%, p=0.04). Overall 30-day mortality was 35%, and was similar between azole-susceptible and non-susceptible groups, with no difference in other outcomes (Table 1). After adjusting for age and hospital-onset, critically ill patients with azole-non-susceptible candidemia had odds ratio of 1.5 (95% CI 0.87 – 2.7 p=0.13) for 30-day mortality. Distribution of candida species and associated 30-day mortality are found in Table 2.

Demographics and Outcome

**Figure:**
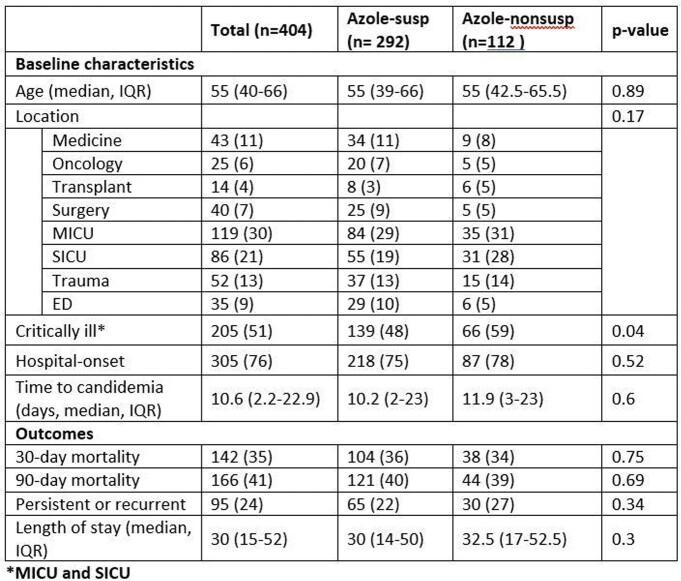

Distribution of most common Candida spp. in candidemia in critically ill (n=205)

**Figure:**
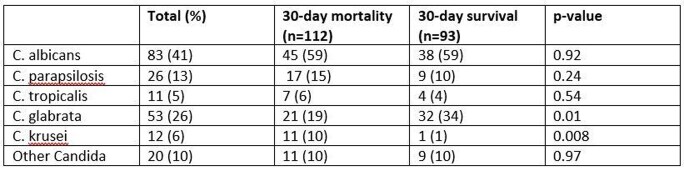

**Conclusion:**

We found similar 30-day mortality rates in azole-susceptible and non-susceptible candida species since echinocandin first-line empiric therapy was implemented in guidelines. The impact on critically ill and invasive infections, including ocular involvement, needs further evaluation.

**Disclosures:**

**All Authors**: No reported disclosures.

